# The environmental bioinorganic chemistry of aquatic microbial organisms

**DOI:** 10.3389/fmicb.2013.00100

**Published:** 2013-04-25

**Authors:** Martha Gledhill, Christel S. Hassler, Veronique Schoemann

**Affiliations:** ^1^National Oceanography Centre, School of Ocean and Earth Science, University of SouthamptonSouthampton, UK; ^2^Plant Functional Biology and Climate Change Cluster, University of Technology SydneyBroadway, NSW, Australia; ^3^Department of Biological Oceanography, Royal Netherlands Institute for Sea ResearchAB Den Burg, Texel, Netherlands

A few key inorganic elements, many of them metals, are essential for life. Approximately 40% of all proteins are metalloproteins which are at the center of the fundamental biological processes that drive biogeochemical cycles. Metalloproteins split water, acquire carbon, reduce carbon, and reoxidize carbon. They are also integral to the nitrogen and oxygen cycles.

In aquatic systems, metals are present at an extraordinarily wide range of concentrations from metal rich hydrothermal systems to the extremely metal poor Southern Ocean. Moreover, the relative abundance of metals to each other is not universal. Such differences are primarily a result of the metal source, input rate, and the major ion (S, O, Cl) composition of their environment. Transition metals, in particular, exhibit diverse environmental behaviors and biological availability, with changes in oxidation state and affinity for non-metals combining to create a rich chemistry and diversity of uses.

It is thus not surprising that this diversity results in a plethora of metal geomes and metal biomes, with organisms exploiting and altering their metallo-environments. A research topic exploring current research themes in environmental aquatic bioinorganic chemistry should thus incorporate articles on a diverse range of subjects. We have been honored to include both reviews and original research articles that taken together, reflect the interdisciplinary nature of the subject area and the diversity of geomes and biomes in which inorganic elements, particularly metals, play a fundamental role.

We have thus been able to include articles on metals, their speciation, and interactions with phytoplankton (Cuss and Gueguen, 2012; Hassler et al., 2012; Shaked and Lis, 2012; Sunda, 2012), on metal acquisition and use by microbes (Barnett et al., 2012; Desai et al., 2012; Glass and Orphan, 2012; Morrissey and Bowler, 2012; Nuester et al., 2012; Scheidegger et al., 2012) and on the effect of inorganic ions in the environment on organism interactions and community structure (Boyd et al., 2012; Gledhill et al., 2012).
